# C-C Motif Chemokine Ligand 2 and Chemokine Receptor 2 in Cardiovascular and Neural Aging and Aging-Related Diseases

**DOI:** 10.3390/ijms25168794

**Published:** 2024-08-13

**Authors:** David Guo, Wuqiang Zhu, Hongyu Qiu

**Affiliations:** 1 Cardiovascular Translational Research Center, Department of Internal Medicine, College of Medicine-Phoenix, University of Arizona, Phoenix, AZ 85004, USA; dguo9@arizona.edu; 2 Department of Cardiovascular Medicine, Physiology and Biomedical Engineering, Center for Regenerative Biotherapeutics, Mayo Clinic Arizona, Scottsdale, AZ 85259, USA; zhu.wuqiang@mayo.edu; 3 Clinical Translational Sciences (CTS) and Bio5 Institution, University of Arizona, Tucson, AZ 85721, USA

**Keywords:** aging, CCL2/CCR2, inflammation, cardiovascular diseases, neural, cancer

## Abstract

Aging is a prominent risk factor for numerous chronic diseases. Understanding the shared mechanisms of aging can aid in pinpointing therapeutic targets for age-related disorders. Chronic inflammation has emerged as a pivotal mediator of aging and a determinant in various age-related chronic conditions. Recent findings indicate that C-C motif chemokine ligand 2 and receptor 2 (CCL2-CCR2) signaling, an important physiological modulator in innate immune response and inflammatory defense, plays a crucial role in aging-related disorders and is increasingly recognized as a promising therapeutic target, highlighting its significance. This review summarizes recent advances in the investigation of CCL2-CCR2 signaling in cardiovascular and neural aging, as well as in various aging-related disorders. It also explores the underlying mechanisms and therapeutic potentials in these contexts. These insights aim to deepen our understanding of aging pathophysiology and the development of aging-related diseases.

## 1. Introduction

Aging is a natural biological process that results in the gradual deterioration of physiological functions over time. This progressive and irreversible process is primarily caused by the accumulation of damage in response to various chronic stressors. Aging significantly increases the prevalence of numerous diseases, particularly cardiovascular and neural disorders, metabolic conditions, and cancers, contributing to high morbidity and mortality among the elderly. Given that aging is a major risk factor for most chronic diseases, understanding the mechanisms underlying the aging process is crucial for identifying therapeutic targets for age-related conditions. Both clinical and basic research have identified several key molecular mechanisms involved in aging, such as chronic oxidative stress, genetic and epigenetic alterations as well as mitochondrial dysfunction. Furthermore, chronic inflammation has emerged as a pivotal mediator of aging and a determinant in many age-related chronic disorders, independent of acute infections [1]. 

Chemokines are a family of cytokines crucial in the migration and recruitment of immune cells within tissues, thereby playing a key role in immune responses. Upon binding to their receptors, chemokines initiate intracellular signaling cascades that promote chemotaxis, degranulation, and actin rearrangement [2,3,4]. One chemokine, chemokine ligand 2 (CCL2), also known as monocyte chemoattractant protein 1 (MCP-1) and small inducible cytokine A2, belongs to the C-C motif chemokine family. CCL2 is expressed in immune cells and other non-immune cells, and its expression can be induced by various mediators including IL-1, IL-4, IL-6, TNF-α, TGF-β, and IFN-γ [5,6]. CCL2 is particularly known for its role in recruiting monocytes and macrophages to sites of inflammation. Although CCL2 can bind to various receptors, C-C motif chemokine receptor 2 (CCR2) is selective for CCL2. 

Chemotaxis has been extensively studied in relation to chemokines such as CCL2. CCL2 serves as a marker for cell transmigration by binding to and activating transmembrane G-protein-coupled receptors, specifically CCR2, on the surface of target cells like macrophages. When CCL2 binds to CCR2, the receptor undergoes a conformational change which activates the associated G-protein. This activation causes the G-protein to dissociate into its alpha (Gα) and beta-gamma (Gβγ) subunits. These subunits then mediate downstream intracellular signaling, including the inhibition of adenylyl cyclase, the activation of phosphatidylinositol 3-kinase (PI3K), and the modulation of small GTPases. These signaling events recruit and activate proteins involved in cell motility and actin polymerization, facilitating cell movement toward the chemokine gradient. Additionally, CCL2-induced activation of CCR2 can increase intracellular calcium levels by activating phospholipase C (PLC), which leads to the release of calcium and promotes the fusion of granules with the plasma membrane. This fusion results in degranulation and the release of superoxide anions. Each of these processes is crucial for the effective functioning of the immune response and inflammation [5,7,8].

Despite initially being studied in innate immune responses and inflammatory diseases, the CCL2-CCR2 axis has garnered significant attention over the past decade for its involvement in aging-related disorders such as cardiovascular disease, cerebrovascular and neurodegenerative diseases, and cancers. Consequently, it is now recognized as a promising therapeutic target [3]. 

Here, we summarize recent findings on the biological function of CCL2-CCR2 signaling in aging processes, highlighting its associations with multiple aging-related diseases and discussing potential interventions and treatments targeting this pathway.

## 2. The Biological Function of CCL2/CCR2 in Immune Response and Inflammatory Defense

The CCL2 gene is located on chromosome 17 in the human genome [7] and comprises three exons and two introns. CCL2 is a monomeric polypeptide with a molecular weight of approximately 13–15 kDa, which can vary depending on the extent of glycosylation [6]. This chemokine is primarily secreted by monocytes, macrophages, and dendritic cells. Platelet-derived growth factor is a major inducer of gene expression. It has been shown that CCL2 exhibits chemotactic activity for monocytes and basophils but not for neutrophils or eosinophils. Additionally, it plays a crucial role in forming granulomas and enhances monocyte anti-tumor activity. Interestingly, the cleavage of CCL2 by matrix metalloproteinase 12 (MMP-12) transforms CCL2 into an agonist for CCR2 [9]. This highlights the remarkable ability of CCL2 to be modified and acquire new functions. This study also indicates that even minor changes in CCL2 can exacerbate disease outcomes by altering signaling involving interacting proteins like MMP-12.

On the other hand, the CCR2 or CD192 (cluster of differentiation 192) gene is located in the chemokine receptor gene cluster region and produces two alternatively spliced transcript variants. The CCR2 protein comprises seven transmembrane domains connected by intracellular and extracellular loops. It features an extracellular N-terminal region crucial for ligand binding specificity and an intracellular C-terminal region involved in intracellular signaling [10,11]. These binding regions are essential for C-C chemokine receptor binding and the activation of the receptor. The N-terminal region of the chemokine is especially important for receptor binding and activation. The chemokine interacts with a shallow groove formed by the N-loop and β3-strand of the receptor, which is essential for its functional activity [12]. In contrast, the C-terminal region is less critical for direct binding but plays a role in the stability and specificity of the receptor. Additionally, the C-terminal region is adaptable, contributing to the dynamic nature of binding and signaling [12].

CCR2 is a functional chemotactic receptor primarily expressed on monocytes/macrophages and lymphocytes and it plays a pivotal role in the biological processes of these cells. By binding to CCL2, CCR2 facilitates the recruitment and migration of monocytes toward the sites of infection or injury. Upon recruitment, the monocytes differentiate into macrophages or dendritic cells depending on the local cytokine environment. These macrophages then play a key role in the inflammatory response by secreting cytokines, engulfing pathogens, and clearing debris. CCR2 can also participate in macrophage polarization. CCR2-positive (CCR2+) monocytes often differentiate into M1 macrophages in inflammatory environments, contributing to inflammation and tissue damage. In certain disease stages, CCR2+ monocytes may adopt an alternative M2 phenotype, which aids in tissue repair and the resolution of inflammation. Although CCR2 is less prominently expressed in lymphocytes compared to monocytes, it can still influence the trafficking of specific lymphocyte subsets, potentially contributing to autoimmune responses and chronic inflammation [13,14]. 

Additionally, CCR2 was identified in various non-immune cells. Located on the external side of the plasma membrane, CCR2 facilitates C-C chemokine binding activity and C-C chemokine receptor activity, influencing processes such as leukocyte migration, cell migration and cytokine production; therefore, it is critical in cellular defense responses, monocyte chemotaxis and neutrophil clearance. CCR2 acts as a high-affinity receptor for several members of the monocyte chemotactic protein (MCP) family, including CCL2, CCL7, CCL8, and CCL13 (human-specific), as well as CCL16 and PC3-secreted microprotein (PSMP) or microseminoprotein (MSMP) [15,16,17,18,19,20]. Among these, the CCL2-CCR2 axis is the most extensively studied and reported. In addition, in vitro studies have shown that CCR2 mediates the clearance of its ligand CCL2 [21], and studies with CCR2 knockout (KO) mice have high levels of circulating CCL2, indicating autoregulatory feedback mechanisms between CCR2 and CCL2 [22]. 

In physiological conditions, the CCL2-CCR2 axis plays a crucial role in stimulating the migration of monocytes during inflammatory responses, which is essential for wound healing [3,4,23]. It is known that, following injury, CCR2+ monocytes are recruited from peripheral blood to wound sites by CCL2. This initiates inflammation, which is later resolved, a process critical for tissue repair. Studies using CCR2 KO mice have shown significantly impaired wound healing at all stages post-injury. This impairment is attributed to reduced pro-inflammatory macrophages and the decreased production of macrophage inflammatory cytokines [23]. However, when monocytes/macrophages from wild-type (WT) mice are adoptively transferred into CCR2 KO mice, normal healing is restored due to the reinstatement of the inflammatory response [23], underscoring the essential role of CCR2 in post-injury wound repair. Similarly, CCR2 KO mice exhibit deficiencies in controlling parasite replication, an excessive influx of neutrophils, and accelerated intestinal damage [24]. These findings further highlight the critical functions of CCR2 beyond wound healing, extending into immune response regulation and tissue homeostasis.

Additionally, the CCL2-CCR2 axis plays a significant role in the inflammatory responses within the central nervous system (CNS). Multiple sclerosis (MS), an inflammatory demyelinating disease of the human CNS characterized by symptoms such as memory loss, impaired thinking, and language difficulties, exemplifies this relationship [25]. In the Experimental Autoimmune Encephalomyelitis (EAE) mouse model, which mirrors brain inflammation and demyelination observed in MS, researchers have observed increased CCR2 expression during peak disease phases, correlating with the severity of MS. Similarly, CCL2 expression levels are linked to disease severity in these models [26]. In support, CCR2 KO mice have shown delayed symptom onset and reduced clinical severity in EAE compared to WT mice. This indicates a detrimental role of CCR2 in MS pathogenesis and suggests there could be potential therapeutic benefits from CCR2 inhibition [26]. 

However, CCR2 activity in CNS inflammation also demonstrates immunomodulatory and protective effects. For instance, CCR2 KO mice in EAE studies exhibit increased neutrophil numbers in lesions, which eventually leads to neuronal demyelination and disease progression at later stages [27]. Moreover, CCR2+ monocytes are proposed as potent regulators of adaptive immunity, suppressing activated T cells post-EAE induction [28]. These findings underscore the dual nature of CCR2 in CNS inflammation, where its effects may vary depending on the stage of leukocyte recruitment [24]. 

On the other hand, CCL2 has been implicated in allergic airway disease (AAD), a chronic inflammatory condition characterized by an excess of T-helper 2 (Th2) lymphocytes in the lower respiratory tract [29]. In AAD, CCL2 promotes Th2 polarization, leading to the release of leukotrienes and histamines, which amplifies inflammation and exacerbates AAD severity [29].

Overall, the CCL2-CCR2 axis is crucial for orchestrating beneficial inflammation during immune responses [4]. However, excessive activation of this axis has been associated with adverse outcomes in MS [26], AAD [29], and cardiac aging [30], highlighting its complex role in various inflammatory conditions.

## 3. CCL2/CCR2 in Aging and Aging-Related Diseases

Aging is often characterized by chronic low-grade inflammation, a phenomenon coined as “inflammaging” [31], which was originally defined as an overall decrease in the ability to respond to different stressors, accompanied by a progressive increase in the organism’s pro-inflammatory status. Inflammaging represents a common systemic physiological process in which there is a progressive increase in pro-inflammatory status and a decline in immune system effectiveness across various cells and organs during advancing age [32]. This chronic inflammation contributes to the accumulation of senescent cells, which further perpetuate inflammation, creating a detrimental cycle [33]. Inflammaging is recognized as a significant risk factor for morbidity and mortality in the elderly, playing a role in the pathogenesis of age-related diseases [34].

Although the mechanisms underlying inflammaging are diverse and mutually non-exclusive, one potential mechanism has been linked to macrophage dysfunction [35,36]. Macrophages, crucial components of the immune system, are responsible for clearing dead cells and combating infections. However, aging compromises macrophage function as they become less efficient in their roles and can become hyperactive. This contributes to sustained inflammation throughout the body. As a result, persistent inflammation in organs can lead to tissue damage and contribute to the development of aging-related diseases [37]. 

Recent studies have highlighted the role of the CCL2-CCR2 axis in inflammaging and its implications in aging-related disorders [38,39]. Below, we discuss key findings related to cardiovascular diseases, neurodegenerative diseases, and other aging-related disorders. 

### 3.1. Cardiac Aging

Cardiovascular disease (CVD) remains a significant global health burden, affecting approximately 523 million people worldwide, with 19 million deaths attributed to CVD annually [40]. These staggering statistics highlight CVD as the leading cause of mortality and morbidity in Western societies [41]. Aging is a crucial independent risk factor for the development and progression of CVD. As individuals age, they are more susceptible to various cardiovascular conditions, such as coronary artery disease, heart failure, and stroke. Age-related changes in the cardiovascular system, including arterial stiffening, endothelial dysfunction, and increased prevalence of hypertension and atherosclerosis, contribute significantly to the morbidity and mortality associated with CVD among the elderly population [42]. 

Cardiac aging refers to the gradual deterioration of the heart’s structure and function over time [43]. This process typically begins with age-related changes such as the thickening of the arterial walls, which increases the strain on the heart by increasing systolic blood pressure and left ventricular afterload [44]. These conditions lead to myocardial remodeling and the stiffening of the heart muscle, reducing its elasticity and impairing its ability to respond to changes in pressure. Consequently, cardiac contractile activity becomes progressively compromised. In addition to structural alterations, cardiac aging involves molecular dysregulation within cardiomyocytes, the cells responsible for heart contraction [45]. Oxidative stress, which intensifies with age, plays a critical role in this process. Increased oxidative stress contributes to interstitial fibrosis (excessive deposition of collagen fibers between cells), contractile dysfunction, and inflammation within the heart tissue [46,47]. These pathological changes disrupt normal cellular function and structure, ultimately leading to cardiac damage and remodeling associated with cardiac aging.

Studies have identified a link between cardiac aging and inflammaging, specifically termed cardiac inflammaging [48,49]. Recent evidence has underscored the causal relationship of the CCL2-CCR2 pathway with cardiovascular disease, both in preclinical models and human studies [39]. In the context of cardiac remodeling, inflammation plays a crucial role in tissue repair. However, excessive inflammation can lead to adverse cardiac remodeling [50]. During the aging process, there is an enhanced inflammatory response [51], characterized by an increased expression of CCL2 in the myocardium [52] and the activation of pro-inflammatory macrophages [3]. 

Research has shown that in the left ventricle (LV) of aging mice, higher concentrations of macrophages are accompanied by increased expressions of CCL2 and MMP-9 [53]. CCL2 plays a pivotal role in promoting MMP-9 expression by recruiting macrophages that express MMP-9. This regulatory relationship is highlighted by the co-localization of MMP-9 expression with macrophages in the LV [53]. Furthermore, MMP-9 levels in the LV correlate with CCL2 levels, indicating an underlying inflammatory mechanism of cardiac aging involving CCL2 and MMP-9 [53]. Notably, age-related inflammation in the LV can be attenuated by the deletion of MMP-9, suggesting a critical role for MMP-9 in mediating inflammatory processes during cardiac aging [54]. The increased inflammation associated with cardiac aging contributes to various effects, such as ventricular hypertrophy, diastolic dysfunction, and valvular degeneration [43]. Moreover, MMP-9’s ability to degrade the extracellular matrix (ECM) underscores its role in age-associated ECM degradation [55]. Together, these findings indicate that CCL2 and MMP-9 are integral to understanding the inflammatory processes involved in cardiac aging, highlighting potential targets for therapeutic intervention aimed at mitigating age-related cardiovascular complications.

In addition to CCL2, CCR2 has also been implicated in aging-related cardiac diseases. The expression of CCR2 on blood monocytes shows a time-of-day–dependent variation, peaking at the beginning of the active phase. Studies have demonstrated that, during the early stages of a cardiac event, elevated CCR2 expression significantly enhances monocyte recruitment to the heart, facilitating their infiltration into cardiac tissue as a response to stress or injury. However, excessive monocyte infiltration exacerbates inflammation, leading to cell death and an increased risk of heart failure (HF) [56]. Studies have shown that the downregulation of CCR2 can mitigate adverse cardiac remodeling, inflammation, and, ultimately HF in animal models [57]. This highlights the pivotal role of CCR2 in mediating inflammatory responses that contribute to cardiac dysfunction.

On the other hand, in animal models such as rats with aortic-cavernous fistula (ACF), CCL2 expression has been observed in cardiomyocytes, vascular endothelial cells, and smooth muscle cells. Furthermore, the severity of congestive HF in these models correlates positively with CCL2 expression levels [39]. It is suggested that CCL2 promotes myocardial damage and dysfunction by binding with CCR2 and activating macrophages. The recruitment of pro-inflammatory macrophages thereby exacerbates cardiac pathology and disease progression [39]. 

**Figure 1:**
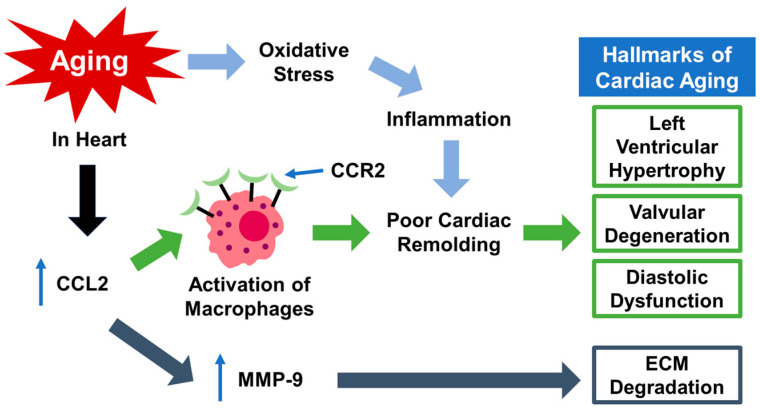
The role and potential underlying mechanisms of CCL2/CCR2 in cardiac aging. This is undertaken through molecular pathways involving aging-induced oxidative stress, the elevated CCL2 that recruits/activates CCR2+ macrophages, and the increased MMP-9. All of these together contribute to cardiac myopathy and dysfunction during aging.

As summarized in Figure 1, these findings underscore the hyperactivity of the CCL2-CCR2 signaling pathway in aging-related heart conditions. The dysregulated recruitment and activation of monocytes and other inflammatory processes mediated by CCR2 and CCL2 contribute significantly to the progression of cardiac dysfunction and HF associated with aging. Targeting this pathway may offer potential therapeutic strategies for mitigating inflammation and improving cardiovascular outcomes in the elderly.

### 3.2. Vascular Aging

Age-related cardiovascular and cerebrovascular diseases often stem from significant alterations in arterial function. During aging, arteries tend to stiffen, develop blockages such as plaques, and become more susceptible to inflammation [58]. These structural changes are accompanied by molecular impacts, including increased degradation of the ECM of the vessels, heightened calcium deposition, and reduced vessel elasticity. These processes lead to intimal thickening, decreased arterial compliance, increased resistance to blood flow, and higher pressure that the heart must work against [44].

Atherosclerosis is one of the most common vascular pathologies associated with aging, characterized by the buildup of plaque within arteries and arterial inflammation [59]. Vascular inflammation is closely linked to the progression of atherosclerosis [60]. Studies have demonstrated that the CCL2-CCR2 axis plays a critical role in promoting inflammation and contributing to the development of atherosclerosis [4]. Notably, there is an increase in CCL2 expression associated with both aging and atherosclerosis [61]. This suggests that aging enhances pro-inflammatory mechanisms, such as CCL2, thereby exacerbating the progression of atherosclerosis. Specifically, increased CCL2 expression promotes the activation and migration of macrophages via the CCL2-CCR2 axis to the sites of arterial plaques during aging [61]. These macrophages contribute to the accumulation of lipids and induce smooth muscle cell proliferation, ultimately forming atherosclerotic plaques [60].

Mechanistic studies have provided insights into how aging affects vascular tissues, particularly through the elevated expression of pro-inflammatory molecules and the activation of macrophages [62]. In aged vessels, endothelial cells (ECs) exhibit increased secretion of pro-inflammatory cytokines such as CCL2 [61]. Additionally, aged aortic vascular smooth muscle cells (VSMCs) show an enhanced expression of CCL2, contributing to arterial wall remodeling associated with aging [30]. In aged mice with atherosclerosis, TGF-β1 upregulates NOX4, which in turn activates CCL2 expression in VSMCs, further linking aging to increased CCL2 expression and inflammation related to atherosclerosis [59]. 

In addition to atherosclerosis, studies highlight the role of CCL2 in age-related vascular changes that contribute to hypertension [63]. Hypertension, characterized by chronically elevated blood pressure in the vessels [64], is closely associated with inflammation [63]. Pro-inflammatory pathways such as CCL2, TGF-β1, and NF-kB intensify the process of vascular aging. Increased CCL2 expression due to aging induces oxidative stress, leading to vascular inflammation and subsequent arterial stiffness, which exacerbates hypertension by increasing the workload on the heart to pump blood [65].

Furthermore, research on mouse models of choroidal neovascularization (CNV) has revealed that advancing age correlates with upregulated pro-inflammatory CCL2-CCR2 signaling, specifically in the choroid but not in the retina. The genetic deletion of CCL2 reduces age-related inflammatory changes in the choroid, leading to decreased recruitment of pro-inflammatory myeloid cells and attenuation of CNV severity. These findings underscore the importance of CCL2 in the recruitment of myeloid cells in exacerbating CNV with age [66].

In summary, CCL2 emerges as a critical mediator in age-related vascular pathologies, contributing to inflammation, arterial remodeling, and complications such as atherosclerosis, hypertension, and choroidal neovascularization.

### 3.3. Neural Aging

As individuals age, various changes occur throughout the body, including in the brain, leading to neural aging characterized by structural and functional deterioration of neural cells in the brain and peripheral nervous system. This process is associated with declines in sensory, motor, and cognitive functions, alongside physical changes such as brain region shrinkage and decreased neuronal communication efficiency. Additionally, cerebral vascular aging can compromise the blood–brain barrier (BBB), increasing its permeability to inflammatory factors and contributing to an inflammatory environment [67]. Clinical studies have shown that monocytes from Alzheimer’s disease (AD) patients have an impaired ability to differentiate into macrophages in vitro compared with monocytes from age-matched controls [68]. Monocytes from aging individuals have been reported to have impaired phagocytosis and increased levels of intracellular tumor necrosis factor-*α*. This suggests that there may be a dysregulation of monocyte function and inflammation in the aging population [69].

Studies have highlighted the role of the CCL2-CCR2 axis in age-related inflammation within the brain. In the central nervous system (CNS), CCL2 is primarily produced by microglia and astrocytes, particularly during pathological conditions [13], while CCR2 is expressed by neurons, astrocytes, and infiltrating leukocytes but not by resident microglia [35,36]. CCL2 functions predominantly by recruiting peripheral inflammatory monocytes expressing CCR2 to lesion sites in the brain, potentially inhibiting long-term potentiation and activating pro-inflammatory pathways via CCR2 on macrophages. This is common in aging, where the inflammatory environment aids in the progression of demyelination and BBB breakdown [70]. Chronic inflammation in the aged brain contributes to neurodegeneration and accelerates age-related brain disorders, often mediated through NF-κB activation in astrocytes, which regulates CCL2 expression and the infiltration of pro-inflammatory factors [67,71]. 

CCL2 and CCR2 signaling is also involved in various aging-related neurological diseases and disorders. AD is a prevalent age-related neurodegenerative disorder characterized by β-amyloid deposition [72]. It has been reported that overexpression of CCL2, driven by glial fibrillary acidic protein promoter, stimulates mononuclear phagocyte (MP) accumulation in the brain, which increases β-amyloid deposition by reducing β-amyloid clearance [73]. Furthermore, the CCL2-CCR2 axis plays an important role in cognitive decline. Elevated CCL2 levels have been associated with brain atrophy and cognitive impairment [74]. Likewise, increased CCL2 expression has also been linked to a faster rate of cognitive decline in AD mice [73]. CCL2 expression promotes monocyte migration and infiltration into the brain in a CCR2-dependent manner. Human studies have shown that higher CCR2 expression correlates with poorer cognitive function, suggesting a direct involvement of CCR2 in human cognition, possibly due to enhanced CCL2 expression in brain microglia and macrophages [75], indicating a link to age-related cognitive decline. 

On the other hand, evidence indicates that CCR2+ cells may play protective rather than detrimental roles in AD. CCR2 KO mice exhibited augmented cognitive impairment and amyloid pathology in an AD model [75,76]. Such features are associated with impaired microglial accumulation, which results in the decreased clearance of *β*-amyloid and increased mortality [75]. Importantly, the transplantation of CCR2-competent bone marrow cells restores cognitive capacity and reduces *β*-amyloid accumulation in CCR2 KO mice after AD [77]. These findings suggest a protective role of CCR2 in the early stage of the disease, whereas CCR2+ monocytes are suggested to be powerful regulators of central nervous system inflammation, contributing to neuronal demyelination and disease progression in later stages.

Collectively, these studies suggest that AD involves dysregulated immune cell responses mediated by CCR2, where the role of CCR2 may shift from protective to detrimental as the disease progresses. The discrepancies observed between mice and humans regarding CCR2’s effects on cognitive function, i.e., CCR2 depletion causing cognitive decline, but higher CCR2 being associated with lower cognitive function, could be attributed to the differing demands for macrophage activation during cognitive decline, particularly in response to increased β-amyloid deposition, a hallmark of AD progression.

### 3.4. Other Aging-Related Disorders

Beyond cardiovascular diseases and neurodegenerative disorders, the CCL2-CCR2 signaling pathway has been implicated in several other aging-related conditions, including chronic inflammation in skeletal muscle, adipose tissue, metabolic disorders, and cancers, as discussed below.

a. Muscular aging

In the context of muscular aging, inflammation emerges as a prominent characteristic, influencing the functionality of muscle tissue over time [78]. Aging muscles exhibit reduced oxidative capacity, which lowers the threshold for triggering inflammatory responses. This altered environment can affect the secretion patterns of macrophages within muscle tissue. Notably, an increased expression of CCL2 in aged muscle suggests a dysregulation in the activation and regulation of muscle macrophages, a process in which the CCL2-CCR2 axis plays a crucial role in recruiting these macrophages during aging [79,80]. 

Recent studies have delved into the impact of inhibiting inflammatory CCR2 signaling on aged muscle regeneration and strength recovery following injury. Researchers have identified CCR2 expression in non-hematopoietic myogenic progenitors (MPs) during muscle regeneration. They observed elevated levels of CCR2 chemokines in regenerating aged muscle, highlighting the necessity of CCR2 deletion in MPs for proper fusion into regenerating muscle tissue. Importantly, after injury, the expression of CCR2 in MPs correlates with levels of its ligands, such as CCL2, CCL7, and CCL8 [38].

In addition, the activation of CCR2 signaling has been found to inhibit MP fusion and its contribution to myofibers, associated with increased MAPKp38δ/γ signaling, phosphorylation of MyoD, and repression of the terminal myogenic commitment factor, Myogenin. Conversely, the timely inhibition of CCR2 post-injury enhances aged regeneration and promotes functional recovery. These findings underscore the detrimental impact of inflammatory-induced CCR2 signaling activation in myogenic cells, contributing to the decline in muscle regenerative capacity associated with aging [38].

b. Cancers

In cancer biology, the CCL2-CCR2 axis plays a crucial role in creating a tumor-permissive microenvironment by promoting tumor-associated macrophage (TAM) infiltration, survival, and activity within the tumor microenvironment (TME). This axis involves CCL2, predominantly produced by peripheral blood mononuclear cells and tumor cells, and CCR2, mainly expressed in monocytes and macrophages. Studies have linked CCR2 expression and CCL2 secretion to poor prognosis in various cancers, including breast cancer, pancreatic cancer, and prostate cancer [75]. The age-related dysregulation of CCL2CCR2 signaling may contribute to increased cancer incidence and progression in older people.

It has been discovered that the interaction between CCL2 and CCR2 is an important signaling axis in cancer growth [51]. CCL2 binding to CCR2 initiates downstream signaling pathways such as JAK/STAT and PI3K/AKT, which activate various transcription factors and genes that promote tumor cell growth and survival [81]. Moreover, CCL2 can establish an immunosuppressive microenvironment within tumors, allowing tumor cells to evade immune surveillance and facilitating tumor proliferation [81]. This immunosuppression is critical for tumor progression and metastasis, as it enables tumor cells to thrive despite the presence of immune cells that would otherwise target and eliminate them.

Additionally, researchers have found that the CCL2-CCR2 axis promotes the progression and metastasis of tumor cells [82]. The activation of the CCL2-CCR2 axis has been closely linked to tumor progression and to the formation of metastases in various cancer types, including breast, colorectal, prostate, melanoma, gastric, and ovarian cancers. One of the mechanisms by which the CCL2-CCR2 axis promotes metastasis is through the recruitment of inflammatory monocytes into the TME. For example, CCL2 increases the expression of MMP-9, a protein that degrades the extracellular matrix and removes physical barriers to tumor metastasis. This process helps explain why the overproduction of CCL2 in cancer cells correlates with enhanced metastatic potential in various cancer types [51]. 

TAMs are a subset of macrophages that reside within the TME and play a critical role in tumor progression. CCL2 contributes to TAM survival during inflammation, which is crucial for maintaining a supportive microenvironment for tumor growth. In models of breast cancer lung metastasis, the activation of macrophages expressing CCR2 by CCL2 leads to the production of CCL3, which in turn mediates TAM retention via CCR1 signaling [51]. This highlights the complex interplay within the CCL2-CCR2 axis in regulating TAM functions and their impact on tumor progression.

**Figure 2:**
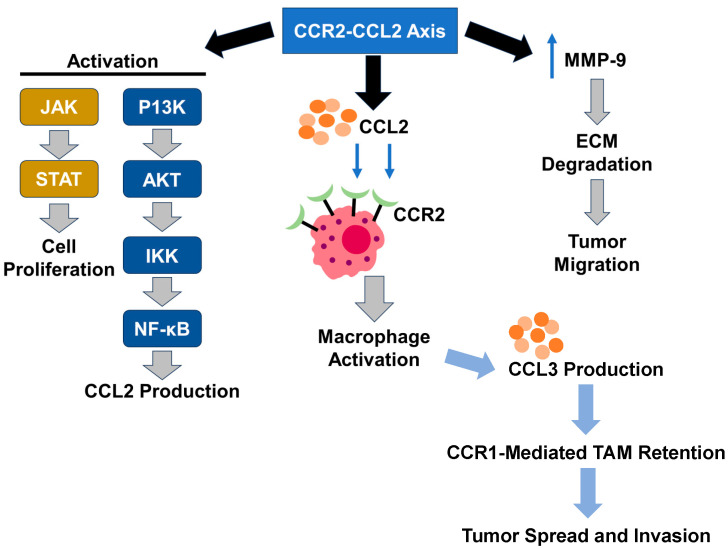
The role and the potential underlying mechanisms of CCL2/CCR2 in cancer development. The CCL2-CCR2 signaling axis participates in cancer development by regulating molecular signaling related to cancer cell proliferation and creating a favorable environment for tumor growth, invasion, and metastasis through activating macrophage inflammatory production as well as MMP-9.

In summary, as illustrated in Figure 2, the CCL2-CCR2 signaling axis in cancer is intricately involved in creating a favorable environment for tumor growth, invasion, and metastasis. Targeting this axis may offer therapeutic opportunities to disrupt tumor-promoting mechanisms mediated by TAMs and reduce cancer progression in affected individuals. Further research into the specific roles of CCR2 and CCL2 in different cancer types and stages of disease progression is crucial for developing effective treatments that can modulate these pathways to benefit cancer patients.

c. Obesity

Obesity, characterized by excessive fat accumulation, is associated with chronic inflammation that significantly impacts health, increasing the risk of conditions like type 2 diabetes and heart disease. Inflammaging can also be driven by an increase in adipose tissue [83]. The CCL2-CCR2 axis, known for its role in inflammatory responses, plays a crucial part in this process [84]. 

Studies in CCR2 KO mice have shown that the absence of CCR2 leads to an increase in eosinophil numbers, macrophage activation, and altered cytokine expression within adipose tissue, particularly under conditions of obesity induced by a high-fat diet [85]. These findings underscore the regulatory role of CCR2 in adipose tissue inflammation and its potential implications in metabolic disorders linked to obesity.

Additionally, the upregulation of CCL2 expression has been associated with cellular senescence in obese adipose tissue. Senescent cells accumulate in adipose tissue along with other senescence-associated factors, including CCL2, contributing to chronic inflammation [86]. This highlights CCL2’s role in promoting inflammation through binding with CCR2 and cellular senescence, further exacerbating metabolic dysfunction in obesity.

In summary, the CCL2-CCR2 axis plays a critical role in promoting chronic inflammation across various tissues and organs affected by aging. This chronic inflammation contributes to the pathogenesis of skeletal muscle deterioration, metabolic disorders like type 2 diabetes, and cancer progression. Targeting this signaling pathway may offer therapeutic opportunities to mitigate inflammation-driven aging-related diseases and improve health outcomes in the aging populations. The studies discussed in this review are also summarized in Table 1.

## 4. Therapeutic Potential by Targeting CCl2/CCR2 Signaling

Given the evidence for the CCL2-CCR2 axis in inflammation and aging, the therapeutic targeting of the CCL2-CCR2 axis has shown promise in mitigating inflammation and potentially treating various aging-related diseases. Here, the present review summarizes key therapeutic strategies and their implications based on recent research.

Deletion and Inhibition of CCL2: Studies have demonstrated that the deletion of CCL2 can reduce macrophage accumulation in conditions such as atherosclerosis. This reduction in macrophage recruitment is beneficial in limiting the progression of inflammatory diseases [87]. In addition, the CCL2 mutant (7ND) is also considered as another alternative inhibiting strategy [39]. This mutant form of CCL2, which lacks the N-terminal seven amino acids, acts as a negative inhibitor by reducing monocyte activation and infiltration. This strategy has shown promise in limiting inflammation and reducing the development of atherosclerotic lesions in animal models [88]. 

CCR2 Inhibition: Therapeutics targeting CCR2 aim to inhibit the receptor’s function, thereby reducing the recruitment of inflammatory monocytes. This approach has effectively reduced CCR2+ monocyte levels in circulation and their infiltration into tissues, such as the heart, in cardiovascular diseases [87]. For instance, studies have shown that CCR2 inhibitors can reduce CCR2 expression and significantly inhibit monocyte recruitment in the heart [87]. Furthermore, the CCR2 antagonist 15a has been shown to reduce circulating inflammatory monocytes and inhibit the development of atherosclerotic plaques in animal models [89]. CCR2 antagonists have also revealed a significant reduction in inflammatory macrophages in the blood and their infiltration into the remodeling myocardium [87]. The decline in inflammatory macrophages reveals the specificity of antagonists on the CCL2-CCR2 axis in this process [90]. These results suggest that targeting chemokines can be a potential therapeutic strategy. 

In addition, many efforts have been made to decrease the activation of CCR2. More recent developments have been made in developing CCR2 agonists to inhibit the development of atherosclerosis. One agonist is INCB3344, which has been reported to reduce the CNS accumulation of monocytes/macrophages in a mice model of EAE [91]. INCB3344 was tested in an apolipoprotein E-deficient (apoE^−/−^) mouse model of atherosclerosis. This CCR2 agonist has been explored for its potential to modulate monocyte trafficking and reduce inflammation. While initial studies showed a reduction in circulating CCR2+ monocytes, its efficacy in reducing atherosclerotic lesions in certain mouse models has been variable and short-lived [92]. Further refinement of agonist therapies is needed to achieve consistent and prolonged therapeutic effects [89].

These therapeutic approaches underscore the critical role of the CCL2-CCR2 axis in driving inflammatory processes associated with aging-related diseases. By targeting this axis, researchers aim to reduce chronic inflammation, mitigate disease progression, and potentially improve outcomes in conditions such as cardiovascular diseases, neurodegenerative disorders, and cancer.

## 5. Conclusions and Future Research Directions

Inflammation has been recognized as a significant endogenous factor in aging, and targeting inflammation may offer a potential anti-aging strategy. CCL2-CCR2 signaling is known to drive the recruitment and activation of pro-inflammatory macrophages, contributing to chronic inflammatory conditions and tissue damage. Recent studies have highlighted the critical roles and potential mechanisms of the CCL2-CCR2 signaling axis in cardiovascular and neural aging, as well as aging-related diseases. 

In cardiovascular conditions, CCL2 and CCR2 are integral to atherosclerosis, myocardial infarction, and heart failure. Elevated CCL2 levels attract monocytes to the sites of plaque formation, where they differentiate into macrophages, contributing to chronic inflammation and plaque instability, potentially leading to myocardial infarction. In addition, CCR2-positive monocytes and macrophages infiltrate the heart tissue, where they exacerbate inflammation and fibrosis. This inflammatory response can impair cardiac function and contribute to the progression of heart failure. 

In the context of neurological aging, elevated levels of CCL2 have also been observed in the brains of individuals with cognitive impairments and in animal models of neurodegenerative diseases such as AD. Increased CCL2 leads to the recruitment of the inflammatory cells into the brain, where they further induce chronic inflammation and neuronal damage. This process is hypothesized to exacerbate the cognitive decline seen in aging.

Understanding the role of CCL2-CCR2 in these conditions helps in developing targeted therapies aimed at reducing inflammation and improving disease outcomes, providing a theoretical foundation for novel and practical anti-aging strategies. However, the therapeutic modulation of CCR2 presents challenges and considerations that must be addressed for safe and effective clinical applications.

(1) Cellular expression beyond monocytes and macrophages: While CCR2 is prominently expressed in monocytes and macrophages involved in inflammation, it is also expressed in other cell types. This broader expression profile implies that any therapy targeting CCR2 could potentially affect these other cell types, which may have diverse functions in different tissues and conditions. Understanding the full spectrum of cell types expressing CCR2 and their roles in various disease contexts is crucial to anticipate unintended consequences of CCR2 modulation therapies. 

(2) Dual role of CCL2-CCR2 axis in diseases: The CCL2-CCR2 axis may play dual roles in diseases depending on the stage and context of the disease process. For instance, in the early stages of inflammation, CCR2-mediated monocyte recruitment may be beneficial for tissue repair. Conversely, excessive or chronic activation of this axis can exacerbate inflammation and contribute to disease progression [36]. Future studies must carefully delineate these dual roles to determine when and how to effectively target CCR2 in aging-related diseases. 

(3) Dynamic nature of monocyte actions: Monocytes are highly dynamic immune cells whose functions can vary depending on their microenvironment. They can exert both pro-inflammatory and anti-inflammatory effects, influencing tissue repair, immune response modulation, and resolution of inflammation. Therefore, understanding the specific roles of CCR2+ monocytes in different disease settings is essential for designing therapies that modulate CCR2 without compromising beneficial immune functions.

(4) Optimizing therapeutic strategies: As research progresses, optimizing CCR2-targeted therapies involves refining the specificity and duration of action of CCR2 inhibitors or agonists. This includes developing strategies to minimize off-target effects on non-immune cells while enhancing therapeutic efficacy in mitigating chronic inflammation in aging-related diseases.

Future Directions: Continued research is needed to elucidate the precise mechanisms by which CCL2-CCR2 signaling contributes to aging-related diseases across various tissues and organs. This knowledge will inform the development of safer and more effective therapeutic interventions targeting CCR2. Moreover, clinical studies are warranted to evaluate the long-term efficacy and safety profiles of CCR2 modulation therapies in aging populations.

In conclusion, while targeting the CCL2-CCR2 axis holds promise for anti-aging strategies by addressing chronic inflammation, the complexity of CCR2 expression and function necessitates careful consideration in therapeutic development. Addressing these challenges through rigorous research will pave the way for novel therapeutic approaches that mitigate inflammation-driven aging-related diseases, ultimately improving health outcomes in the aging populations.

## Figures and Tables

**Table 1:** Summary of the studies of CCR2/CCL2 in aging-related disorders.

Condition	Experimental Model	Conclusion	Reference
Cardiac Aging	C57/BL6J mice	Increased CCL2 and MMP-9 expression in senescent mice is seen in the same locations as macrophages. This indicates an underlying inflammatory mechanism of cardiac aging.	[53]
Vascular Aging	LysM-Cre/MyD88-flox, CD11c-MyD88 Tg and SM22α-Cre/MyD88-flox mice	Aging increases the production of CCL2 in VSMCs which play a role in arterial wall remodeling associated with aging.	[30]
Atherosclerosis	LysM-Cre/MyD88-flox, CD11c-MyD88 Tg and SM22α-Cre/MyD88-flox mice	Aged cells have increased CCL2 levels, which results in the accumulation of macrophages in plaques in atherosclerosis.	[61]
Hypertension	Male Fisher 344 rats	Aging-associated increases in CCL2 lead to vascular inflammation, increased central blood pressure, and ultimately hypertension.	[65]
Choroidal Neovascularization (CNV)	CCL2-knockout C57Bl/6 mice	CCL2 exacerbates CNV with age through the recruitment of myeloid cells.	[66]
Neural Aging	Immortalized human BMEC line hCMEC/D3	Aging leads to the deterioration of the blood–brain barrier (BBB), which creates an inflammatory environment.	[67]
Cerebrovascular Aging	SAMP1 mice	In the aged brain, the CCL2-CCR2 axis activates pro-inflammatory pathways and accelerates age-related brain disorders.	[70]
Alzheimer’s Disease (AD)	CCR2 Knockout mice	There is increased CCL2 expression in AD. Higher CCR2 expression leads to a decreased cognitive function, indicating a link to age-related cognitive decline.	[75]
Muscular Aging	SCID Mice	Aging causes impaired activation and regulation of muscle macrophages and higher CCL2 expression.	[79]
Cancer	Human non-small cell lung cancer cell line A549	The CCL2-CCR2 axis leads to downstream signals that suppress the immune system and activate tumor cell growth.	[81]

## Data Availability

Not applicable.
